# High prevalence of porcine cysticercosis in slaughtered pigs in Rwanda: An abattoir survey

**DOI:** 10.1371/journal.pntd.0012598

**Published:** 2024-10-25

**Authors:** Anselme Shyaka, Nadine Rujeni, Eric I. Kanyamibwa, Geofrey Kagabo, Eric M. Fèvre, Rupert J. Quinnell

**Affiliations:** 1 School of Biology, Faculty of Biological Sciences, University of Leeds, Leeds, United Kingdom; 2 Center for One Health, University of Global Health Equity, Butaro, Rwanda; 3 School of Health Sciences, College of Medicine and Health Sciences, University of Rwanda, Kigali, Rwanda; 4 Institute of Infection, Veterinary and Ecological Sciences, University of Liverpool, Liverpool, United Kingdom; 5 International Livestock Research Institute, Nairobi, Kenya; IRNASA, CSIC, SPAIN

## Abstract

Porcine cysticercosis (PC) is an important public health problem, especially in sub-Saharan Africa, but limited information is available on the prevalence of infection in pigs entering the food chain. Existing diagnostic methods vary in accuracy and efficiency; whole carcass dissection is the most reliable method but is labour-intensive and destroys the carcass so can only be used in a research setting. Serological tests offer lower specificity, while meat inspection and lingual examination lack sensitivity, hampering accurate estimates and the removal of infected pigs from the food chain. Here, we provide the first estimates of PC prevalence in abattoirs in Rwanda. We use whole carcass dissection to determine the diagnostic accuracy of a commercial antigen-ELISA to estimate the true prevalence of infection across Rwanda and identify *Taenia* species affecting local pigs. We carried out a cross-sectional survey in 6 abattoirs across Rwanda (n = 744 pigs), with whole carcass dissection of a subset of 67 pigs. Cysts were detected in 20/67 (30%) of carcasses, with >1000 cysts in 9/20 (45%) of infected pigs. All cysts were identified as *Taenia solium* by PCR-RFLP, with no cysts of *Taenia hydatigena* found. The antigen-ELISA showed a sensitivity of 90% (95% CI: 68–99) and specificity of 85% (95% CI: 72–94), when compared to dissection. Using these estimates, the true prevalence was calculated as 25–43% in two abattoirs in south-west Rwanda, and 2–3% in the rest of the country. Fewer than half of infected pigs were detected by tongue palpation and post-mortem veterinary inspection. Our data indicate a high prevalence of PC in Rwandan abattoirs. Tongue palpation and veterinary inspections, as currently carried out, have little impact in removing cyst-infested pigs from the food chain. Additional interventions are needed, such as proper pig husbandry, treatment and vaccination against cysticercosis, health education, improved sanitation and hygiene, and improved processing and cooking of meat.

## Author summary

Porcine cysticercosis is a tapeworm infection with a complex life cycle involving pigs and humans. This parasite is the cause of over a third of cases of acquired epilepsy in regions of the world where sanitation is poor and free-range pigs are reared, and is a public health and economic challenge to consumers and farmers. Our study aimed to estimate the prevalence of tapeworm infection in slaughtered pigs in Rwanda, in order to support disease control. We found that porcine cysticercosis is widespread across the country with a high prevalence in the south and south-west. Pigs with very high numbers of tapeworm cysts were identified and constitute a public health concern to consumers. We showed that the locally available tests for infection such as tongue palpation and veterinary meat inspection are not efficient at preventing infected pigs from reaching consumers. Control of cysticercosis in Rwanda will require further interventions that support hygienic pig production practices and improved pork processing and cooking, ensure appropriate on-farm treatment to stop transmission of porcine cysticercosis and human taeniasis and increase community awareness and behaviour change.

## 1. Introduction

Cysticercosis is a zoonotic disease caused by infection with the metacestode larval stage of *Taenia solium* and is associated with a heavy public health and economic burden in endemic areas of Africa, Asia and South America [1]. The adult tapeworm lives in the human intestine and is acquired from the consumption of undercooked pork containing cysticerci of *T*. *solium*. Pigs get infected with larval stages when they eat human stools containing eggs excreted by a tapeworm carrier, or food or water contaminated with *T*. *solium* eggs. Humans can also be infected with larval stages through accidental ingestion of tapeworm eggs, causing human cysticercosis, which can evolve into neurocysticercosis (NCC) when cysts migrate into the central nervous system. Cysticercosis has been recognized as the most important foodborne parasitic disease globally [2] and is associated with high disease burden and stigma for epileptic persons in the case of NCC. However, WHO considers its control possible through the integration of various available tools, including (but not limited to) adequate meat inspection and processing [3].

There are a variety of diagnostic methods available for the detection of *T*. *solium* in pigs [4]. The gold standard is whole carcass dissection to detect cysts in pig tissues, but this is time-consuming and expensive, and as it destroys the carcass can only be used for research purposes [5]. Tongue palpation, which is commonly practiced in Rwanda [6], has a low sensitivity of only 10–21% [7,8] and so may only detect pigs with a very high burden of infection [9], although it has value as a means of estimating the presence of infection in a population of pigs [10]. Post-mortem inspection of carcasses by veterinarians also has a low sensitivity of 22–39%, especially in carcasses with a low burden of infection [7,11,12]. Serological tests to detect either antibodies or circulating antigens have much higher sensitivities but have lower specificity. The monoclonal antibody-based B158/B60 Ag-ELISA has been used in several surveillance and epidemiological studies in Africa and elsewhere [7,8,13–15]. The reported sensitivity (65–91%) and specificity (68–97%) compared to carcass dissection vary considerably between studies [7,8,13,15]. Some of the low specificity is due to cross-reactivity with other *Taenia* species such as *T*. *hydatigena* [16]. *T*. *hydatigena* has a low but variable prevalence in African pigs, with estimates varying from 3–19% [8,17–19], but there are no data from Rwanda.

Rwanda, like several of its neighbouring countries, has a fast-growing pig production industry, currently dominated by smallholder farmers. Pigs are raised for relatively rapidly available agricultural income and to produce manure for crop production. This increase in smallholder pig production and pork consumption in Africa has been linked to the emergence of porcine cysticercosis [20]. There is very little published data on cysticercosis prevalence in Rwanda, but the prevalence of cysticercosis in patients with epilepsy in southern Rwanda is high [21]. Two localised studies have shown that meat inspection detects cysticercosis in 4–20% of carcasses in some areas of Rwanda [22,23]. This shows a potential high risk of cysticercosis infection in pork that enters the food chain, but no studies have been performed using more sensitive techniques.

In the present study, we investigated porcine cysticercosis (PC) in pigs slaughtered at recognised pig abattoirs in Rwanda to determine the country level prevalence of porcine cysticercosis in pigs entering the food chain from the formal pork supply system. The objectives were to: a) to determine the burden and species of *Taenia* in pigs in Rwanda, (b) to determine the diagnostic accuracy of a commercial B158/B60 Ag-ELISA kit in Rwanda, in comparison to carcass dissection, and (c) to use Ag-ELISA to estimate the country-wide prevalence of PC in known formal abattoirs, with estimates adjusted for the diagnostic accuracy of the test.

## 2. Materials and methods

### 2.1 Ethics statement

Ethical clearance was obtained from the Rwanda National Ethics Committee (Reference 165/RNEC/2017) and from the University of Leeds (UK) Faculty of Biological Sciences Research Ethics Committee (Reference BIOSCI 16–019).

### 2.2 Study area and design

**Fig 1:**
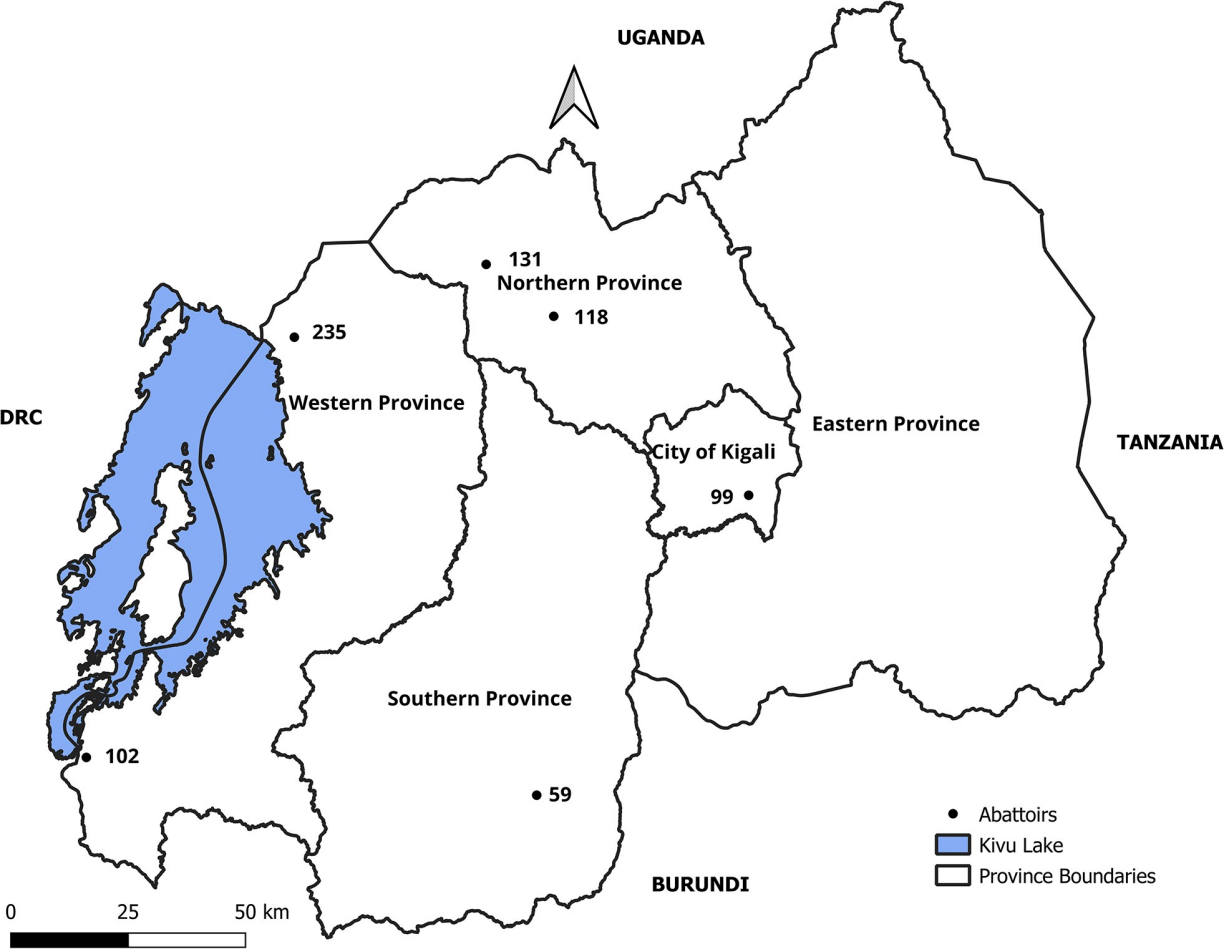
Map of Rwanda showing approximate location of sampled abattoirs (black filled circles) and the number of pigs sampled at each abattoir. (QGIS Ver. 3.28—CC-BY license 4.0.). The layers are freely accessible from https://diva-gis.org/data.html and can be shared under CC-BY license 4.0.

We have previously identified 8 abattoirs in 6 different locations of Rwanda that operate regularly in the formal sector [6]. Here we sampled 6 abattoirs: 2 in Western Province, 2 in Northern Province, 1 in Southern Province and 1 in Kigali (Fig 1). The other 2 abattoirs (in Western Province) were not operating during the sampling period. These represent all the areas where pig farming forms a significant part of livestock production. The abattoirs belonged to local government (n = 2), private owners (n = 3) or were under the management of a cooperative (n = 1). The abattoirs were supplied by a diversity of sources, including local live pig markets or farms, through a network of farmers, butchers, pig traders and middlemen. The abattoir pig and pork supply chains are described elsewhere [6].

### 2.3 Pig data and blood sample collection

On sampling days, every pig brought to the abattoir that day was recruited into the study. For each pig, the breed, sex, and presence/absence of external parasites (fleas and/or ticks) was recorded. The general body condition was evaluated by visually assessing how well the bone structure (ribs, hip bones, and backbone) was covered by the body fat [24]. The tongue was inspected by pig brokers before the slaughter took place, to look for the presence of cysts–this is routinely done in pig markets in Rwanda to help determine the selling price. A blood sample was collected immediately on slaughter from the jugular vein, in a plain collection tube in which blood was allowed to clot. Samples were centrifuged and the serum extracted and stored at -20°C until analysis. Finally, routine veterinary meat inspection was done by the official meat inspectorate, according to FAO guidelines [25]. Briefly, the external carcass surfaces and oral and nasal cavities were checked. Multiple incisions were then made in the head lymph nodes, masseters, diaphragm, abdominal muscles and tongue to check for cysts. The viscera were inspected, the heart opened and a deep incision made in the septum. Finally, several other lymph nodes were excised and inspected. To reduce the risk that our presence might influence the inspection process, we were not present in the same room during the inspection. We recorded the decision made by the veterinary inspector on whether the whole carcass or individual organs were condemned and the reason for condemnation. From sample size calculations for estimating prevalence, a minimum of 97 pigs were needed per site for a precision of 10%, at a 95% confidence level and assuming a conservative prevalence of 50%. The actual number sampled from one abattoir was lower due to the low numbers of pigs being slaughtered at that site at the time.

### 2.4 Carcass dissections

To determine the Ag-ELISA performance and confirm the presence and morphology of cysts, 67 pigs were sampled for carcass dissection from two abattoirs: Gisagara (Southern Province, n = 47 pigs) in a high prevalence area and Musanze (Northern Province, n = 20 pigs) in a low prevalence area. These pigs were a subset of those sampled for the country-wide survey; this was a convenience sample, with 2–4 pigs awaiting slaughter bought at the market price each day. Pigs were chosen without knowledge of the tongue palpation results and prior to meat inspection. Carcass dissections were carried out as described by Chembensofu *et al*. [8]. Briefly, after slaughter and removal of the skin, carcasses and heads were kept refrigerated until dissection (generally for 24–96 h). Before dissection, the surfaces of the viscera and the intraperitoneal cavities were searched for cysts, including *T*. *hydatigena* cysts. Then, muscles from the half carcass of each pig were excised from the bones and cysts enumerated in the half carcass and in the complete head (including brain and eyes), tongue, neck, heart, lungs, diaphragm, psoas muscles, spleen, liver, and kidneys. The muscles/organs were sliced thinly (maximum 5 mm thickness) to identify and count the cysts.

The total number of cysticerci per pig was determined as follows: if cysticerci were identified in the first half carcass, then the total number of cysticerci was calculated as twice the number of cysts in the first half carcass added to the count of the head, tongue, neck, heart, lungs, diaphragm, psoas muscles, spleen, liver, and kidneys. If no cysticerci were found in the first half, the other half was dissected and the total count was determined as the count in the second half, plus the counts in the head and internal organs as above. In one case, the cysticerci were too numerous to count. For this pig, the whole carcass (minus internal organs and the head) was weighed and 1 kg of muscle was excised from the forelimb and hindlimb, dissected and cysts enumerated [13]. The total number of cysts in this pig was estimated as the count in the dissected muscle, adjusted to the carcass weight, plus the full counts of the head and internal organs as above. Cyst viability was evaluated by assessing the presence of translucent fluid with a visible whitish protoscolex. Cysts were considered non-viable in the absence of the cystic fluid or when the cysticercus wall was viscous, collapsed, or damaged. In addition, non-cystic, yellowish structures were considered calcified and thus non-viable [11,12].

### 2.5 PCR and Restriction enzymes

Cysticerci were collected from muscles and organs of the positive carcasses for confirmation by PCR-restriction fragment length polymorphism (PCR-RFLP). DNA from at least one cyst from muscle and another organ (brain, heart, tongue, diaphragm) of each infected pig was analysed. Genomic DNA was extracted using the DNeasy Blood and Tissue Extraction Kit following the manufacturer’s instructions (QIAGEN, Hilden, Germany). The extracted DNA was used in PCR to amplify a mitochondrial 12S rDNA gene fragment with the primer pair TaenF, 5’ GTTTGCCACCTCGATGTTGACT 3’ and ITMTnR, 5’CTCAATAATAATCGAGGGTGACGG 3’ described by Geysen *et al*. [26]. Each amplified PCR product was then subjected to a double digestion using *Dde*I and *Hinf*I in a single tube containing CutSmart buffer (New England Biolabs) [27] as well as a single digestion in a separate tube using *Hpa*I in CutSmart buffer (New England Biolabs) as described by Devleesschauwer *et al*. [28]. The digested fragments were separated by agarose gel electrophoresis (2% GelRed agarose LE (Biotium, USA) in 45mM Tris-Borate, 1mM EDTA, pH 8.3). DNA restriction fragments were visualized using an Azure Biosystems 280 Gel documentation system (Azure Biosystems, Dublin, CA, USA).

### 2.6 ELISA

ELISAs were carried out using a commercial antigen detection ELISA kit (apDia Turnhout, Belgium) designed to detect circulating *T*. *solium* antigen. Porcine serum samples were tested in duplicate, following the manufacturer’s instructions. Briefly, each serum sample was pre-treated using the provided 5% trichloroacetic acid buffer and then neutralized using the neutralization buffer. This resulted in a final dilution of 1:4 for the samples and controls. The neutralized samples were added in coated microtitre strips and incubated on a shaker (800 rpm) for 15 minutes at 37°C. After incubation, sample wells were washed 5 times using the washing buffer and excess liquid was removed by tapping the plate onto absorbent paper. Then, 100 μl of conjugate solution was added and samples incubated for 15 minutes (37°C, 800 rpm) followed by a washing step as described above. Next, 100 μl of chromogen solution was added to each well and sealed strips incubated for 15 minutes at room temperature in the dark. After incubation, 50 μl of stop solution was added and the optical densities (ODs) measured at 450 nm in a microplate spectrometer (iMark, Bio-Rad). According to the manufacturer’s instructions, two negative control samples were included on each plate, and results for each serum were expressed as signal/negative ratios relative to the mean of the negative controls on that plate. OD values that were higher than the maximum detection limit (OD = 3.5) were assigned that value.

### 2.7 Statistical analysis

Carcass dissection was used as the gold standard to estimate the diagnostic accuracy of the Ag-ELISA, with sensitivity and specificity calculated using the *epiR* package in R version 4.1.1 [29]. The optimum cut-off was confirmed from ROC analysis using the *cutpt* command in Stata 19; the Youden, Liu and nearest to (0,1) methods all gave the same result. Spearman’s rank correlation coefficient was applied to ascertain the association between ELISA values and the number of cysts. The ELISA results for each abattoir were first expressed as apparent prevalence (AP), that is the number of ELISA-positive pigs tested divided by the total number of pigs tested. Differences in AP according to pig characteristics were tested by contingency table Pearson chi-squared analysis. AP was then adjusted to true prevalence (TP) estimates considering the ELISA test sensitivity (Se) and specificity (Sp) established in this study. This was done by applying the Rogan & Gladen equation [30] using the *epiR* package or, in situations where the AP is less than (1-Sp), by using Bayesian methods to take into account low prevalence scenarios as described by Messam et al. [31]. Briefly, the number of Ag-ELISA positive pig samples is **x | (Se, Sp, TP) ~ binomial (n, AP)** where **AP = Se × TP + (1—TP) (1—Sp).** To estimate the true prevalence of cysticercosis in low AP situations (which was the case for most abattoirs, except two located in southern regions of Rwanda), prior distributions for Se and Sp and estimates from this study were used. The *betaPERT* function of the package *prevalence* [32] was used to model Beta distributions for Se and Sp based on available information. Hence the Se was modelled to be between 0.65 and 0.91 with our observed value of 0.90 as the most likely value, whereas the Sp was modelled to lie between 0.68 and 0.97 with 0.851 as the most likely value [7,8,13,15]. Due to the lack of available estimates for Rwanda, the posterior true prevalence was estimated assuming uninformative priors for the true prevalence and as such the priors for the TP were uniform beta (1,1). Markov Chain Monte Carlo (MCMC) methods were used to obtain posterior estimates for TP and the model was run for 200,000 iterations, with the first 10,000 burn-in results discarded. The posterior distributions and the 95% posterior credible interval (95% PCI) are reported as the median and 0.025 and 0.975 quantiles of the posterior distribution of TP.

## 3. Results

### 3.1 Characteristics of pigs sampled

A total of 744 pigs were sampled in 6 abattoirs across Rwanda: Rubavu (n = 235), Musanze (n = 131), Gakenke (n = 118), Rusizi (n = 102), Kigali (n = 99) and Gisagara (n = 59) (Fig 1 and Table 1). Most of the pigs were cross breeds between local and various exogenous breeds, with lower numbers of Landrace, Large White, local breeds and Pietrain. More pigs were female than male, a high proportion of pigs had ectoparasites such as fleas and/or ticks while fewer had sores and/or vesicles on the skin (Table 1).

**Table 1:** Characteristics of Pigs Sampled.

Variables		Frequency (%)
Abattoir	Gisagara	59 (7.9)
	Kigali	99 (13.3)
	Musanze	131 (17.6)
	Gakenke	118 (15.9)
	Rubavu	235 (31.6)
	Rusizi	102 (13.7)
Pig Breed	Cross	391 (52.6)
	Landrace	139 (18.7)
	Large White	97 (13.0)
	Local/indigenous	99 (13.3)
	Pietrain	18 (2.4)
Pig Sex	Male	315 (42.3)
	Female	429 (57.7)
General condition	Overly Fat	23 (3.1)
	Fat	307 (41.3)
	Ideal	347 (46.6)
	Thin	67 (9.0)
Presence of ectoparasites	Yes	327 (44.0)
	No	417 (56.0)
Type of ectoparasite	Fleas	205 (62.7)
	Ticks	25 (7.6)
	Fleas and Ticks	97 (29.7)
Presence of sores/vesicles	Yes	118 (15.9)
	No	626 (84.1)
Gender of Person accompanying the pig	Male	527 (70.8)
	Female	217 (29.2)
Person accompanying the pig	Butcher	15 (2.0)
	Farmer	27 (3.6)
	Middleman	80 (10.8)
	Pig/Pork Trader	622 (83.6)

### 3.2 Pig dissections

Cysts were identified in 20 of the 67 dissected pigs (29.9%): 20/47 pigs (42.6%) from Gisagara and 0/20 pigs from Musanze. The total number of cysts found varied from 1 to 12,032 with a median of 254 cysticerci. Among the dissected pigs with cysticerci, only 5 pigs had light infection intensities (<100 cysticerci), while 9 pigs had heavy infection intensities (>1000 cysticerci), of which two pigs had more than 10,000 cysticerci (Table 2). All cysticerci were macroscopically consistent with *T*. *solium* cysticerci.

**Table 2:** Pig carcass infection intensities.

Number of cysticerci in carcass	Number of carcasses (%)	Number of Pigs positive by tongue palpation	Number of Pigs detected at meat inspection
<100	5 (25)	2	1
101–1000	6 (30)	3	1
1001–10000	7 (35)	3	5
>10000	2 (10)	2	2
Total	20	10	9

Precise counts were performed in 19 pigs. These pigs had 32,062 viable cysticerci and 106 non-viable cysticerci, representing 99.7% and 0.3% of all cysticerci respectively; all pigs had at least one viable cyst. Dissection results showed higher tissue infestations with cysticerci in predilected sites such as fore and hind quarters and masseter. Cysticerci were found in the brain, heart and diaphragm but no cysticerci were found in the liver, spleen, stomach or intestines (Table 3).

**Table 3:** Distribution and stage of *Taenia solium* cysts detected in different organs in 19 dissected pigs.

Organ /Muscle	Cysticercus stage		Total (%)
	Viable	Non-viable	
Brain	145	0	145 (0.5)
Tongue	1211	0	1211 (3.8)
Head	3165	6	3171 (9.9)
Neck	1048	0	1048 (3.3)
Heart	906	90	996 (3.1)
Diaphragm	602	0	602 (1.9)
Forequarter	8816	4	8820 (27.4)
Hindquarter	7195	6	7201 (22.4)
Psoas	1561	0	1561 (4.9)
Liver, Spleen, Lung, Kidney, Stomach, Intestines	0	0	0 (0.0)
Others	7413	0	7413 (23.0)
Total	32062	106	32168 (100)

### 3.3 PCR and restriction enzymes

**Fig 2:**
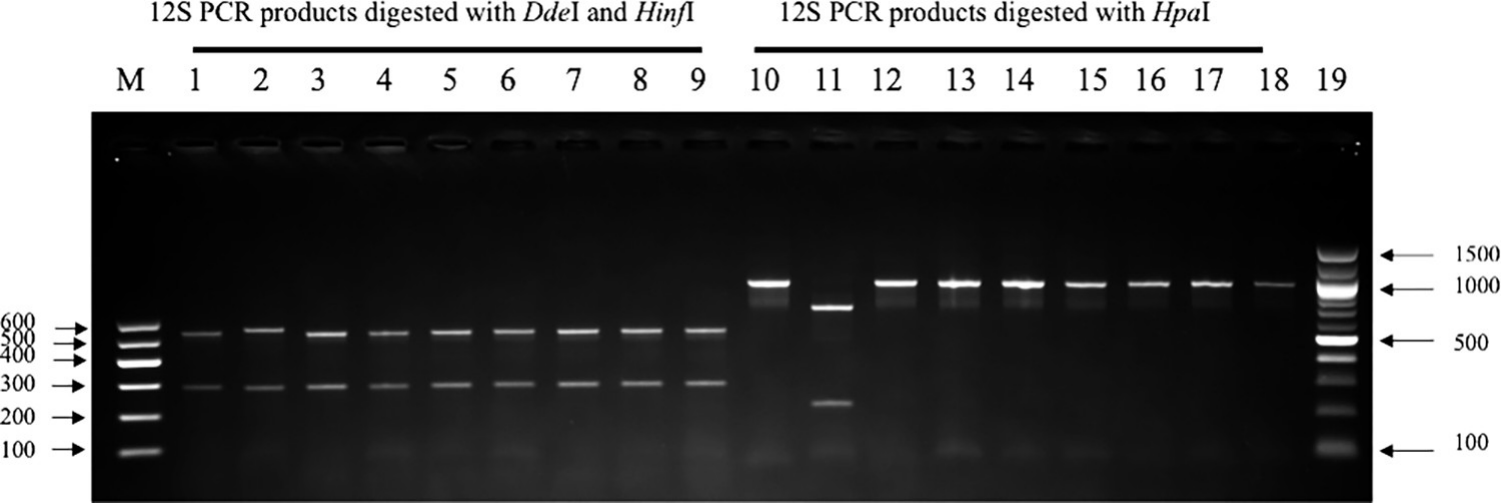
PCR-RFLP of DNA extracted from cysts recovered from pig dissections in Rwanda. Lanes (3,12), (4,13), (5,14), (6,15), 7, 16), (8,17), (9,18) are digested PCR products from this study, derived from the same DNA in the case of each pair. Lanes (1, 10) and (2,11) contain *T*. *solium* and *T*. *hydatigena* DNA controls. Lanes (M, 19) are 100 bp DNA Ladders. All study samples show the expected fragment sizes for *T*. *solium*.

All cysticerci collected from various organs of the 20 dissected positive pigs were morphologically identical. For each pig, DNA from 1–3 cysts was analysed from different tissues, according to availability. A total of 41 cysticerci was tested, 22 from muscle, 11 from diaphragm, 6 from tongue and 2 from brain. All the analysed cysticerci were confirmed by PCR-RFLP as *T*. *solium* (Fig 2): all samples showed the expected bands of around 300bp and 550bp after *Dde*I/*Hinf*I digestion, which distinguishes *T*. *solium/T*. *hydatigena* from *T*. *saginata*, *T*. *asiatica* and *Echinococcus granulosus*, and no samples were digested by *Hpa*I, which identified them as *T*. *solium* not *T*. *hydatigena* [33].

### 3.4 Comparison of ELISA and dissection data

Sera from all dissected pigs were tested by Ag-ELISA. The manufacturer’s suggested cut-off for a positive result is a s/n ratio of 3.5, which was confirmed by ROC analysis. Using this cut-off, 25/67 dissected pigs (37%, 95% CI: 27–49%) tested positive on Ag-ELISA, of which 18 had cysticerci of *T*. *solium*, whereas 7 had no detected cysticerci. All pigs that were negative for cysts, including these 7, had undergone full carcass dissection. Two dissected pig carcasses with viable cysticerci tested negative on ELISA (Table 4). The Ag-ELISA had a sensitivity of 90.0% (95% CI: 68.3–98.8) and specificity of 85.1% (95% CI: 71.7–93.8). The positive and negative predictive values were 72% and 95% respectively.

**Table 4:** Results of Ag-ELISA and carcass dissection on the 67 dissected pigs.

		Dissection		
		Positive	Negative	Total
**B158/B60 Ag-ELISA**	Positive	18	7	25
	Negative	2	40	42
	Total	20	47	67

There was a strong relationship between the number of cysts found and the magnitude of the Ag-ELISA ratio across all dissected pigs (rho = 0.72, *p* < 0.001) and across the subset of 27 pigs that were positive in either test (rho = 0.65, *p* < 0.001). Seventeen pigs had high ELISA ratio values (>35), and of these 15 were cyst-positive (PPV = 88%), while only 3 of 8 antigen-positive pigs with lower s/n ratios were cyst positive (PPV = 37.5%). This higher cut-off had a specificity of 96%, but a sensitivity of 75%. Tongue palpation detected 10/20 cyst-positive pigs and no cyst-negative pigs, a sensitivity of 50% (95% CI: 27–73) and specificity of 100% (95% CI: 93–100). This method had positive and negative predictive values of 100% and 82% respectively. Routine meat inspection detected 9/20 cyst-positive pigs (and no cyst-negatives), giving a sensitivity of 45% (95% CI: 23–69) and a specificity of 100% (95% CI: 93–100). This method had positive and negative predictive values of 100% and 81% respectively.

### 3.5 ELISA results on abattoir samples

Out of 744 pig blood samples collected in 6 abattoirs across Rwanda, 116 tested positive on Ag-ELISA, an apparent prevalence (AP) of 15.6% (95% CI: 13.2–18.4). AP varied across sampled abattoirs, with high values in abattoirs located in the south and south-west (47.5% and 33.3% respectively in Gisagara and Rusizi). The remaining abattoirs had relatively low seropositivity values of 8.1–10.7% (Table 5). The proportions of positive Ag-ELISA were statistically different across sampled abattoirs (χ^2^  =  88.2, df = 5, *p* < 0.0001) but did not vary significantly by pig sex (χ^2^  =  2.94, df = 1, *p* = 0.09) or pig breed (Fisher’s exact test, *p* = 0.051). Seventy-two of the 116 positive sera were strongly positive, with a s/n ratio >35, including some sera from each abattoir.

**Table 5:** Results of Ag-ELISA on all sampled pigs.

Area	AP (pos/total)	TP (95% CI)	Strong positive AP (pos/total)	Positive tongue	Positive Vet. Inspection
Gisagara	47.5 (28/59)	43.4 (27.1–60.0)	28.8 (17/59)	11 (18.6)	11 (18.6)
Rusizi	33.3 (34/102)	24.5 (13.4–37.3)	23.5 (24/102)	7 (6.9)	12 (11.8)
Musanze	10.7 (14/131)	3.2 (0.1–11.5)	6.9 (9/131)	0 (0)	0 (0)
Kigali	10.1 (10/99)	3.2 (0.1–12.0)	3.0 (3/99)	1 (1)	0 (0)
Gakenke	9.3 (11/118)	2.8 (0.1–10.4)	5.9 (7/118)	0 (0)	0 (0)
Rubavu	8.1 (19/235)	1.8 (0.1–6.9)	5.1 (12/235)	0 (0)	0 (0)

True prevalence values were estimated from the APs by adjusting for the test specificity and sensitivity calculated in the previous section. The TP could be calculated directly for the two abattoirs with high AP, while Bayesian estimates were derived for the abattoirs with low prevalence (Table 5). True prevalence was 43% and 25% in Gisagara and Rusizi respectively, and 2–3% in the other abattoirs.

Of the 744 pigs, 19 (2.6%) had cysts on the tongue detected whereas 23 (3.1%) pigs were found to have cysts by the veterinarian at meat inspection. Comparing the percentages positive to the true prevalence in Gisagara and Rusizi gives an approximate sensitivity of 28–43% for tongue examination and 43–48% for veterinary inspection. The Ag-ELISA detected 17 of the 19 (89.5%) tongue-positive pigs and 21 of the 23 pigs (91.3%) detected by the veterinary inspectors. Of the 23 infected pigs detected by the veterinarian, 7 were totally condemned (meat withdrawn from food chain). For the 16 other pigs, individual organs were condemned: heart (n = 7), tongue (n = 1), both heart and tongue (n = 7) or heart, tongue, and whole head (n = 1).

## 4. Discussion

In this study, we estimated the prevalence of *T*. *solium* in pigs slaughtered for human consumption at 6 abattoirs across Rwanda. This study focussed on formally recognised abattoir facilities in the country to target pigs reaching a geographically widely distributed market chain, including urban areas. Our study shows that infection in pigs was present in all sampled abattoirs, but with pronounced geographical variation. The two abattoirs in the south-west of the country had very high prevalences of 25–43%, while prevalences in the rest of the country were lower (2–3%). To the best of our knowledge, this is the first national survey of porcine cysticercosis prevalence at official abattoirs in Rwanda. Importantly, these estimates reflect infection that enters the food chain and provide insights on the risk of human cysticercosis/neurocysticercosis in Rwanda. Only an estimated 43–48% of infected pigs were detected by meat inspection, which emphasises the risk to consumers.

We assessed pig infection using the Ag-ELISA, which is increasingly used for epidemiological surveys. The sensitivity and specificity of the assay vary between studies, so we derived our own estimates for the commercial kit, comparing the test to carcass dissection, considered as a gold standard [8]. The estimated sensitivity (90%) and specificity (85%) were comparable to the sensitivity and specificity estimated in other studies of 65–91% and 68–97% respectively [7,8,13,15]. Ag-ELISA methods demonstrate an ongoing infection and are more sensitive than tongue palpation especially in detecting recent and light infections [34–36]. The high sensitivity in the current study may reflect a relatively high intensity of infection, and a high proportion of viable cysts, as the probability of detection is known to increase with viable cyst burden [8]. We detected circulating antigen in 7 pigs which had no cysticerci at dissection (false ELISA positives). Various hypotheses have been formulated for this known lack of specificity. Such pigs may represent early infections, since antigens of *T*. *solium* are produced several weeks before full development of cysticerci, or aborted infections in which infection does not lead to the development of mature cysticerci, or light infections with only one or a few cysts present, which could be missed during necropsy [37]. False positives may also result from cross-reactions with antigens of other tapeworm species, especially *T*. *hydatigena*. *T*. *hydatigena* has often been assumed to be rare in Africa, but recent dissection or carcass inspection studies have shown a prevalence of 3–19% in a range of countries [8,17–19,38]. Here, we found no evidence of *T*. *hydatigena* infection, so cross-reaction is unlikely to have been a problem. The absence of this species may be due to the low numbers of dogs (the definitive host) in Rwanda [39].

The estimated true prevalence values reported here indicate that porcine cysticercosis is highly prevalent in two of the sampled abattoirs, which represents a significant threat to public health. Prevalence figures in Rwanda are comparable to those reported in other countries in the region: one study in Zambia found a high prevalence of 56% [8], with lower prevalence in Uganda of 8.6–16.2% [40,41] and Kenya of 4.4% [42]. The variation in the figures across the region may be associated with different risk factors for porcine infection by *T*. *solium*. We have not cascaded our results into a comprehensive food chain risk assessment [43], but we consider that an unacceptable infection pressure is present in the pork system in Rwanda, and that investment is merited to reduce this risk and protect the health of the pork consuming public.

The prevalence in Rwanda varied markedly geographically. Our data suggest a division of the sampled regions into two distinct areas: the south and south-west region with very high true prevalence (25–43%) and the remainder of the country with relatively low values (true prevalence of 2–3%). Up to now, data on the prevalence of PC in Rwanda have been scarce. A recent study carried out in Southern Province [22], estimated a prevalence of 9.2% at slaughter using tongue palpation which reduced to 4% at veterinary inspection. Given the sensitivity of the two methods used by the study, the true prevalence is certainly higher, as shown here. The higher risk in the southern regions doubtless relates to husbandry techniques, which include open grazing and free roaming of pigs inside and outside the homestead.

The prevalence of PC is lower in abattoirs in Kigali and the north and north-west, supplied by a network of brokers that buy pigs directly from farms, including semi-intensive and intensive production systems located in north and east parts of Rwanda [6]. However, infections are still present at all sites, as shown by the TP estimates and the presence of pigs with high antigen levels. The fact that pigs in the northern parts of the country are mainly kept in pens and are not allowed to roam freely inside and outside the household reduces the probability of ingesting eggs of *T*. *solium*. However, many pigs are fed on grass and crop residues collected in the field and in forests, which might have been contaminated by eggs of *T*. *solium* [6,44]. In addition, even in these semi-intensive systems, young animals are often allowed to roam inside and around the households, increasing the possibility of contact with contaminated faeces. Lastly, it is common for all the slaughterhouses to source some slaughtered pigs from highly endemic southern areas [6]. In particular, many pigs slaughtered in Rusizi are sourced from southern markets.

In addition to the formal abattoirs, much pig slaughtering in Rwanda is carried out informally in the backyards of farms, butcheries and restaurants. These slaughter places are characterized by lack of adequate veterinary inspection and may attract pigs rejected at market level, following a positive tongue palpation for cysticercosis detection. This is most likely the case because pigs suspected of having cysts following tongue palpation lose their economic value at live pig markets or abattoirs, and are often taken back to the farm for backyard slaughter and local consumption [6,22]. This suggests that there might be differences in infection rates between formal and informal sectors; given this, our estimates representing the formal value chain could be considered conservative, with the overall porcine cysticercosis prevalence likely to be higher.

Nevertheless, two risk trajectories emerge from this work. Firstly, local consumption of animals on farms presents a risk to farming households and for propagation of the *Taenia* life cycle where pork is consumed, and pigs are kept. Secondly, trade in pigs and geographically distant consumption of pork presents risks to populations who purchase pork but do not raise pigs, such as urban residents. A similar pattern of risk has been noted in other countries in the region [14]. There is clearly a risk of transmission of *T*. *solium* associated with pork consumption in the broader national pork food chain, though this risk will be mitigated to some extent by cooking and consumption habits [45]. Future studies should investigate to what extent the risk is mitigated and reduced before it reaches the pork consumers since the current tongue palpation and post-mortem meat inspection are not sufficiently sensitive.

There is a need for behavioural change in the Rwandan pig farming industry to tackle cysticercosis. Focus must be put on structural determinants that drive people’s compliance with prevention measures of zoonotic diseases [46], and multisectoral interventions need to be deployed to address the *Taenia* life cycle at multiple stages: veterinary services, access to education and investment, as well as provision of efficient treatment and prevention options for cysticercosis must be strengthened. In particular, the availability of an infection-blocking vaccine for cysticercosis [47] should be a major consideration for a co-ordinated cysticercosis control action, as long as the mode of distribution and uptake can be well planned in consultation with pig farmers [48].

Effective screening of pigs at slaughter to prevent infection entering the food chain would be a valuable control measure. Meat inspection is known to have a low sensitivity and so misses a high proportion of infected pigs. Here the sensitivity of meat inspection was slightly higher than in other studies, an estimated 43–48%. It is possible that our presence at the abattoir increased the rigour of the meat inspection process, leading to a higher sensitivity, or this may reflect high average cyst burdens. Heavily infected pigs were more likely to be detected by meat inspection, as previously reported elsewhere [7], which means that meat inspection will detect a higher proportion of cysts than pigs. However, a major concern with meat inspection in Rwanda is that positive tests are often not followed by whole carcass condemnation. A more sensitive test point-of-slaughter test than meat inspection would be useful. The Ag-ELISA, whilst more sensitive, is not suitable for routine use in abattoirs as it requires technical expertise, expensive equipment and reagents and takes time. However, development of a cost-effective rapid test for antigen detection would be very valuable [49].

*T*. *solium* cysticercosis is a public health challenge that requires the involvement of actors from various disciplines in a One Health approach. In practice, public health efforts could be directed to treat taeniasis cases and ensure the management of human cysticercosis. At the same time, veterinary services must strengthen pig farming, focusing on proper pig feeding and adequate husbandry. Local leaders and the community should be involved in efforts to improve WASH and knowledge, attitudes, and practices towards *T*. *solium* infections, such as the need for effective cooking of pork.

## 5. Conclusion

Our study has shown that porcine cysticercosis is prevalent in all sampled abattoirs in Rwanda, representing all geographical regions of the country where pig production takes place. There are, however, geographical variations in the level of risk, most likely linked to the production systems from which pigs originate. The Ag-ELISA used in this study requires careful interpretation given issues of sensitivity and specificity, but it is a valuable tool for national surveillance of this nature where data are otherwise lacking on risks associated with transmission of the parasite. With a burgeoning pig industry and pork consumption in Rwanda, cysticercosis needs to be considered as a core component of the national NTD control plan.
